# An Integrated Approach of Bioassays and Non-Target Screening for the Assessment of Endocrine-Disrupting Activities in Tap Water and Identification of Novel Endocrine-Disrupting Chemicals

**DOI:** 10.3390/toxics12040247

**Published:** 2024-03-28

**Authors:** Siyuan Liu, Jing Liu

**Affiliations:** 1 MOE Key Laboratory of Environmental Remediation and Ecosystem Health, College of Environmental and Resource Sciences, Zhejiang University, Hangzhou 310058, China; 2 Institute of Environmental Health, College of Environmental and Resource Sciences, Zhejiang University, Hangzhou 310058, China

**Keywords:** drinking water, endocrine-disrupting chemicals, bioassay, non-target analysis, steroid hormone receptors

## Abstract

The safety of drinking water is a significant environmental issue of great concern for human health since numerous contaminants are often detected in drinking water and its sources. Boiling is a common household method used to produce relatively high-quality drinking water in some countries and regions. In this study, with the aid of an integrated approach of in vitro bioassays and non-target analysis based on high-resolution mass spectrometry coupled with liquid chromatography, alterations in endocrine-disrupting activities in tap water samples without and with boiling were revealed, as well as the potential endocrine-disrupting chemicals (EDCs) contributing to these alterations were identified. The organic extracts of tap water had no significant (ant)agonistic activities against an estrogen receptor (ER), progesterone receptor (PR), glucocorticoid receptor (GR), and mineralocorticoid receptor (MR) at enrichment concentrations of ≤10 times, posing no immediate or acute health risk to humans. However, the presence of agonistic activities against PR and MR and antagonistic activities against ER, PR, GR, and MR in OEs of tap water at relatively higher enrichment concentrations still raise potential health concerns. Boiling effectively reduced antagonistic activities against these steroid hormone receptors (SHRs) but increased estrogenic and glucocorticoid activities in drinking water. Four novel potential EDCs, including one UV filter (phenylbenzimidazole sulfonic acid, PBSA) and three natural metabolites of organisms (beta-hydroxymyristic acid, 12-hydroxyoctadecanoic acid, and isorosmanol) were identified in drinking water samples, each of which showed (ant)agonistic activities against different SHRs. Given the widespread use of UV filters in sunscreens to prevent skin cancer, the health risks posed by PBSA as an identified novel EDC are of concern. Although boiling has been thought to reduce the health risk of drinking water contamination, our findings suggest that boiling may have a more complex effect on the endocrine-disrupting activities of drinking water and, therefore, a more comprehensive assessment is needed.

## 1. Introduction

Drinking water safety has long been a significant environmental issue of great concern for human health. Numerous contaminants, such as heavy metals, pesticides, antibiotics, and industrial organic chemicals, are commonly detected in drinking water and its sources [1,2,3,4,5]. Many of these contaminants are reported to be endocrine-disrupting chemicals (EDCs), which pose potential risks to ecological systems and human health through water consumption [6,7].

Human exposure to drinking water contaminants typically involves a combined pollution of multiple chemicals at low concentrations [8]. As a traditional water quality testing method, targeted chemical analysis is unlikely to capture all the chemicals, especially at low concentrations, and may ignore the possible combinatorial effects of complex mixtures of pollutants. For example, while some individual pollutants are weakly toxic at environmental levels and their health risks are negligible, the presence of their mixtures can have adverse effects on ecosystems and humans and their health risks can no longer be ignored [9,10]. A battery of in vitro bioassays have emerged as a sensitive and reliable method to determine the toxic or endocrine-disrupting effects of individual chemicals or mixtures of all active chemicals in environmental samples [11,12]. For a more comprehensive water quality assessment, some studies have applied bioassays to assess endocrine-disrupting activity in drinking water and its sources [13,14,15]. However, most of these studies have focused on estrogenic and androgenic activities, while other hormonal pathways such as progesterone, glucocorticoid, and mineralocorticoid have been less well studied, although they also play important roles in reproduction, development, and metabolism [16]. Therefore, a broader assessment of multiple endocrine activities in drinking water is necessary.

Non-target analysis based on high-resolution mass spectrometry, coupled with liquid or gas chromatography, treats a signal characterized by exact masse and retention time as a distinct feature and tracks features based on spatial, temporal, and process changes in a given context, thus allowing for the identification of unknown chemicals through exact mass, isotope, adduct, and fragmentation information without the need for prior information [17,18,19]. Non-target analysis allows for the simultaneous detection of thousands of known and unknown compounds in an environmental sample and has been applied to identify unknown chemicals in water [20,21,22]. Recent research has expanded the utility of non-target analysis by integrating it with in vitro bioassays. This integrated approach has been applied to evaluate the efficacy of treatment methods for wastewater, reuse water, and drinking water [23,24,25]. Therefore, integrating non-target analysis with in vitro bioassays could be a valuable method of assessing water quality and identifying potential EDCs in drinking water.

Boiling is the most commonly used method of water treatment in some countries or regions, such as China [26]. The effectiveness of boiling for pathogen inactivation and contaminant removal has been evaluated [27,28,29]. Some studies have found that boiling alters the composition of disinfection by-products in drinking water [30,31]. However, the impact of boiling on the endocrine-disrupting activity in drinking water remains unclear.

In this study, the endocrine-disrupting activities in tap water and boiling water were assessed by detecting the (ant)agonistic activities against an estrogen receptor (ER), progesterone receptor (PR), glucocorticoid receptor (GR), and mineralocorticoid receptor (MR). To further understand the impact of boiling on water quality, the characteristics of non-target features in tap water and boiled water were analyzed and potential EDCs were further identified in drinking water. This integrated approach enables a comprehensive understanding of the health risks associated with drinking water contamination and the effectiveness of boiling in mitigating these risks.

## 2. Materials and Methods

### 2.1. Chemicals and Reagents

Dimethyl sulfoxide (DMSO) and 17β-estradiol (E_2_; purity, >97%) were purchased from Sigma-Aldrich (St. Louis, MO, USA). Progesterone (PRO; 100%), spironolactone (SPI; >97%), and tamoxifen (TAM; ≥99%) were purchased from Selleck Hem (Houston, TX, USA). Cortisol (HC; >98%), 2,6-ditert-butyl-4-nitrophenol (DNP; ≥98%), bentazone (BEN; analytical standard), and beta-hydroxymyristic acid (3-HTA; ≥98.0% (GC)) were bought from Aladdin Reagent (Shanghai, China). Mifepristone (RU486; >98%) was obtained from Tokyo Chemical Industry (Tokyo, Japan). Aldosterone (ALD; >99%) was purchased from J&K Scientific Ltd. (Beijing, China). Dodecylbenzene sulfonic acid (DBSA; 95%) was obtained from Macklin (Shanghai, China). Phenylbenzimidazole sulfonic acid (PBSA; ≥98%) and 12-hydroxyoctadecanoic acid (12-HA; ≥97.0%) were purchased from Med Chem Express (Shanghai, China). Isorosmanol (ISO; ≥98.0%) was obtained from Tan-Mo Technology Co., Ltd. (Changzhou, China). 2-Naphthalene sulfonic acid (2-NSA; ≥98.0%) was purchased from Meryer (Shanghai, China). These chemicals were dissolved in DMSO and stored at −20 °C. Information on the 43 external standards with a wide range of physicochemical properties used to evaluate the extraction process of water samples is listed in Supporting Information (SI) Table S1. These standards were dissolved in methanol and stored at −20 °C. All solvents and reagents used in this study were HPLC grade and purchased from J&K Scientific Ltd.

### 2.2. Preparation and Extraction of Water Samples

In October 2022, 120 L of a pooled water sample was collected from one tap at 12 separate times in Hangzhou, Zhejiang province. The water sampling containers were glass bottles that were washed with detergent and water, then rinsed with ultrapure water, and then with methanol. In total, 60 L of water (20 L for non-target screening and 40 L for bioassays) was boiled using an electric kettle, which has a built-in top to reduce the potential for post-boiling contamination. Another 60 L of water was untreated. Samples were stored in the dark at 4 °C until extraction.

The enrichment and concentration of organic pollutants were accomplished using solid phase extraction (SPE), as described previously with modification [32,33]. The organic extracts (OEs) of drinking water samples were extracted using the Oasis HLB extraction cartridge (500 mg, 6 mL) (Waters, Milford, MA, USA), which had been preconditioned with methanol and ultrapure water. Each SPE cartridge was designed to filter 5 L of water. After the tap water samples flowed through the cartridge at a rate of 5~7 mL/min, the cartridge was then eluted with 12 mL methanol: acetone (*v*/*v*, 7:3) at a rate of 3~5 mL/min. The eluates were combined, transferred to a rotary evaporator, and concentrated to <1 mL. The concentrated extracts were evaporated to near dryness under a gentle nitrogen flow and then reconstituted in 100 μL DMSO to achieve an enrichment factor of 400,000× for bioassays or in 500 μL methanol for non-target screening.

### 2.3. Cell Culture

The Chinese hamster ovary K1 (CHO-K1) cell line used in this study was obtained from the National Collection of Authenticated Cell Cultures (Shanghai, China). CHO-K1 cells were maintained in Dulbecco’s modified eagle medium (DMEM) (Hyclone, Logan, UT, USA) supplemented with 10% fetal bovine serum (FBS) (Hyclone) and 100 U/mL streptomycin-penicillin (Hyclone) under the condition of an atmosphere of 5% CO_2_ at 37 °C with saturating humidity.

### 2.4. MTS Assay

The cytotoxicity of OEs to CHO-K1 cells was determined using MTS assays. CHO-K1 cells were treated with OEs concentrated 1, 10, 50, 100, 200, 300, and 400-fold relative to the actual water samples or 0.1% DMSO (as vehicle control) for 24 h. The final concentration of DMSO in the medium did not exceed 0.1%. The cell viability was measured using CellTiter 96^®^ AQ_ueous_ One Solution Cell Proliferation Assay (Promega, Madison, WI, USA) as previously described [34]. The concentration of formylhydrazine expressed as absorbance measured at 490 nm with a microplate reader (Infinite M200 PRO, Tecan, Switzerland) was proportional to the number of viable cells.

### 2.5. Dual-Luciferase Reporter Gene Assays for ERα, PR, GR, and MR

The rat ERα expression plasmid rERa/pCI and estrogen responsive element (ERE) containing reporter plasmid pERE-AUG-Luc+ are kind gifts from Dr. M. Takeyoshi (Chemicals Assessment Center, Chemicals Evaluation and Research Institute, Oita, Japan) [35]. Human PR expression plasmid pSG5-hPR is a kind gift from Dr. Pierre Chambon (Institute for Genetics and Cellular and Molecular Biology, Illkirch-Graffenstaden, France) [36]. Human GRα expression plasmid pF25GFP-hGRα and reporter plasmid pMMTV-luc that contains progesterone responsive element (PRE), glucocorticoid response element (GRE), and mineralocorticoid response element (MRE) are generous gifts from Dr. Evangelia Charmandari (Biomedical Research Foundation of the Academy of Athens, Greece) [37]. Human MRα expression plasmid pEGFP-C1-hMRα is a kind gift from Dr. Claudia Grossmann (Universität Halle-Wittenberg, Halle, Germany) [38]. pRL-TK (Promega, Madison, WI, USA) was used as an internal control in the dual-luciferase reporter assays according to the manufacturer’s instructions.

CHO-K1 cells were seeded in 96-well plates in phenol red-free DMEM with 5% FBS for 24 h overnight before plasmids transfection. For detection of ERα, PR, GR, and MR activities, cells were transfected with expression plasmids for these four steroid hormone receptors (SHRs) and the plasmids containing their corresponding responsive elements using lipofectamine 3000 reagent (Invitrogen, Frederick, MD, USA), as previously described [12]. After 4 h of transfection, cells were replaced with fresh phenol red-free DMEM without serum overnight. The next day, cells were exposed to 0.1% DMSO (as vehicle control), OEs, or reference compounds. After 24 h of exposure, firefly and renilla luciferase activities were measured using the Dual-luciferase Reporter Assay Kit (Promega, Madison, WI, USA) on a fluorescence spectrophotometer (Infinite M200 PRO, Tecan, Männedorf, Switzerland), as previously described [34]. The relative transactivation of each steroid receptor was presented as the ratio of firefly to renilla luciferase activity. Dose–response curves were plotted to quantify the agonistic activity responses using steroid hormones as reference compounds: E_2_ for ERα, PRO for PR, HC for GR, and ALD for MR. The agonistic activities of SHRs in OEs of water are shown as corresponding steroid hormone equivalents ng/L water, i.e., E_2_ equivalent (EEQ) for ERα, PRO equivalent (PEQ) for PR, HC equivalent (HEQ) for GR, or ALD equivalent (AEQ) for MR.

To measure the antagonistic activity of SHRs, cells were pretreated with OEs or reference SHR antagonist for 30 min and then treated with 10^−9^ M E_2_ or 10^−6^ M PRO or 10^−8^ M HC or 10^−10^ M ALD, respectively, in combination with OEs or the corresponding SHR antagonist, as described previously [12]. Dose–response curves were plotted to quantify the antagonistic activity responses using the SHR antagonists as reference compounds: TAM for ERα, RU486 for PR and GR, or SPI for MR. SHR antagonistic activities in OEs of water were calibrated against reference SHR antagonist and expressed as the corresponding equivalents ng/L water or μg/L water, i.e., TAM equivalent (TEQ) for ERα, RU486 equivalent (REQ) for PR and GR, or SPI equivalent (SEQ) for MR.

Agonistic and antagonistic equivalents for SHRs were calculated as described previously with modifications [39]. EEQ, PEQ, HEQ, AEQ, TEQ, REQ, and SEQ of the tested water samples were derived by dividing the concentration of corresponding reference compounds that achieved 20% of its maximum effect (*EC*_20_) by enrichment factors of the tested water samples that produced an equivalent response (Equation (1)).

(1) $$Equivalents=\frac{{EC}_{20}\;of\;reference\;compounds}{enrichment\;factors\;of\;tested\;samples}$$

### 2.6. Non-Target Screening

Non-target screening of the water samples was performed by electrospray ionization-Q-TOF-MS/MS method using AB Triple TOF 6600^+^ system (AB SCIEX, Framingham, MA, USA) coupled with ultraperformance liquid chromatography (UPLC) (Waters Corp., Milford, MA, USA). Three microliter organic extract in methanol was injected onto an ACQUITY UPLC CSH C18 column (1.7 μm, 2.1 × 150 mm; Waters Corp.) oven temperature of 50 °C. Mobile phases A and B were 0.1% formic acid-water and 0.1% formic acid-acetonitrile, respectively. The linear gradient programs were as follows: 0/5, 5/5, 23/35, 31/95 (min/B%). The flow rate was 0.3 mL/min. The maximum allowed error was set to ±5 ppm. Positive and negative ionization (PI and NI) modes were operated with a source voltage of +5.5 kV and −4.5 kV as well as a source temperature of 550 °C and 550 °C, respectively. The collision energy was set at 40 V ± 20 V. The scan ranges of *m*/*z* of precursor ion and product ion were set as 100−1500 Da and 50−1500 Da, respectively. The exact mass calibration was performed automatically before each analysis employing the Automated Calibration Delivery System.

The raw data were first converted into the Analysis Base File format using the free software Reifycs Analysis Base File Converter ver 1.3 (https://www.reifycs.com/abfconverter/ (accessed on 10 March 2023)). Then, peak picking, alignment, filtering, and identification of all non-target features were accomplished using an open-source software MS-DIAL ver 4.9 (http://prime.psc.riken.jp/compms/msdial/main.html (accessed on 10 March 2023)) [40]. The MS-DIAL automatically achieved the compound identification by calculating the similarity of accurate mass, isotope ratio, retention time, and MS/MS spectrum with the reference database MassBank (http://www.massbank.jp (accessed on 11 March 2023)), which contains over 500,000 mass spectral records of 15,000 chemicals. Only features with a peak high of at least 1000 amplitudes and a response at least 10 times higher compared to the control (procedural blank sample) were considered. The MS-DIAL outputs a table including features such as accurate *m*/*z*, retention time similarity, total score, and intensity (reported as peak area), etc.

### 2.7. Quality Assurance and Quality Control for OEs Extraction and Chemical Analysis

A total of 43 external standards including phthalates esters, phenols, organophosphorus insecticides, neonicotinoid insecticides and their metabolites, pyrethroid metabolites, and perfluorochemicals were spiked into ultrapure water at a concentration of 50 ng/L to test the recovery of aqueous sample extraction. As shown in Figure S1, the recovery rates of these organic chemicals ranged from 42% to 146%. A total of 86% of these organic chemicals were detected within the normal range of recoveries (from 65% to 125%), which proves that the method employed could retain most compounds. Three procedural blank samples (ultrapure water) were extracted via SPE and analyzed along with the samples. No other target organic chemicals were detected in the blank samples. Three duplicate samples were prepared to ensure the repeatability of the entire process.

### 2.8. Data Analysis

In bioassay experiments, each sample was tested at least 3 times independently in a 96-well plate with 4 replicate wells for each concentration. The IBM SPSS Statistics 26.0 (SPSS, Chicago, IL, USA) and Origin 2023b (OriginLab, Northampton, MA, USA) were used for statistical analysis. Data for all experiments were shown as the mean ± standard deviation (SD). *p*-Values < 0.05 are considered “statistically significant” (*), while <0.01 are viewed as “statistically highly significant” (**).

## 3. Results and Discussion

### 3.1. Agonistic and Antagonistic Activity against OEs of Tap Water in ERα, PR, GR, and MR Assays

To determine the impact of the OEs of water with or without boiling on the SHR-mediated activity, CHO-K1 cells transfected with SHR-luciferase reporters were exposed to OEs at concentrations equal to 1–400 times the actual concentrations in tap water or boiled water. As shown in Figure S2, the selected concentrations of OEs had no significant cytotoxicity to the CHO-K1 cells.

**Figure 1:**
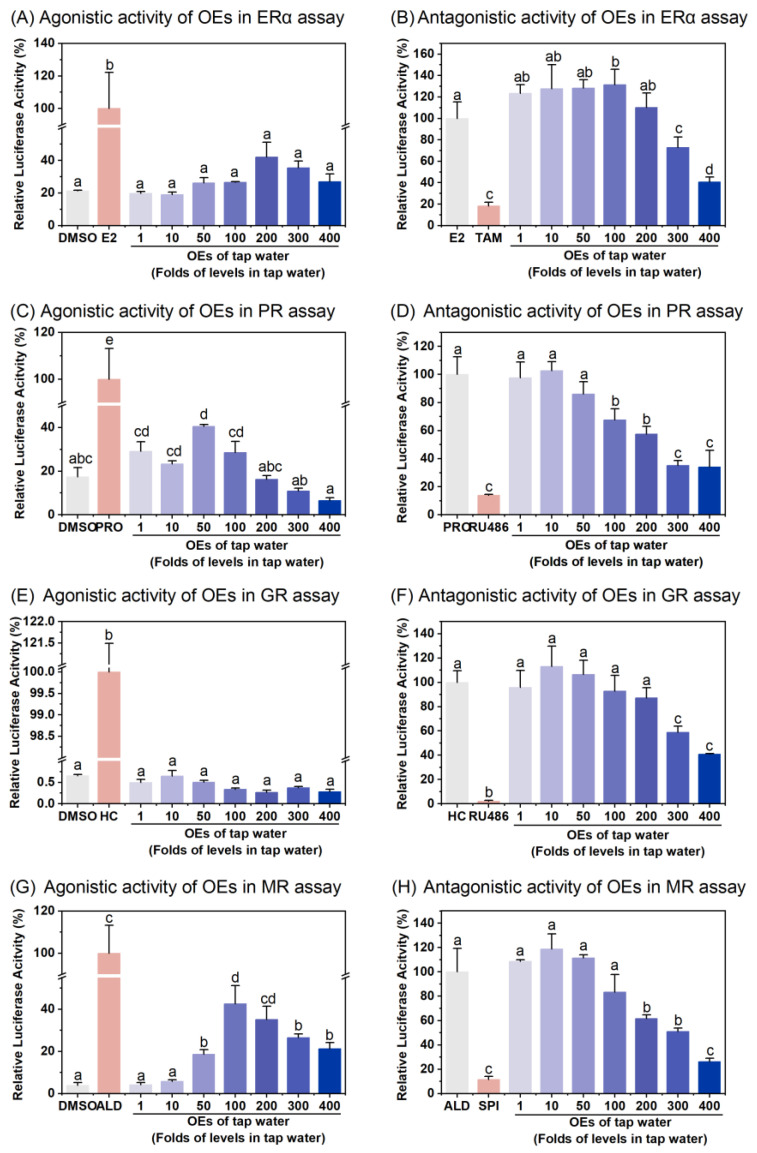
Agonistic and antagonistic activity of organic extracts (OEs) of tap water in ERα, PR, GR, and MR assays. Transfected CHO-K1 cells were treated with 0.1% DMSO (as vehicle control), 10^−9^ M E_2_ or 10^−6^ M PRO or 10^−8^ M HC or 10^−10^ M ALD (as positive control), or OEs of tap water for agonistic effects in bioassays for ERα (**A**), PR (**C**), GR (**E**), and MR (**G**), respectively. Transfected CHO-K1 cells were treated with 10^−9^ M E_2_ or 10^−6^ M PRO or 10^−8^ M HC or 10^−10^ M ALD (as positive control), the combination of corresponding steroid hormone with 10^−5^ M TAM or 10^−7^ M RU486 or 10^−7^ M RU486 or 10^−4.5^ M SPI (as antagonist control), or the combination of corresponding steroid hormone with OEs of tap water for antagonistic effects in bioassays for ERα (**B**), PR (**D**), GR (**F**), and MR (**H**), respectively. Results were expressed as percent induction, with 100% activity defined as the activity achieved with positive control. Data are shown as mean ± SD (*n* = 4). Different letters on bars indicate significant differences among treatments (*p* < 0.05).

Table 1 shows the agonistic and antagonistic equivalent of OEs on SHRs. Specifically, the OEs of tap water did not induce significant ERα transactivation at any tested concentrations (Figure 1A), suggesting no detectable estrogenic activity in tap water samples. Conversely, the OEs significantly attenuated E_2_-induced ERα transactivation at 300- and 400-fold enrichment concentrations in a dose-dependent manner (Figure 1B), indicating anti-estrogenic potential, with a TEQ of 4.53 μg/L water (Table 1). The agonistic activity against OEs of tap water in PR assays increased and then decreased, reaching a maximum and statistical significance at a 50-fold enrichment concentration (Figure 1C), with an equivalent value of 43.84 ng PEQ /L water (Table 1). The antagonistic effects of OEs on PR increased with increasing enrichment concentrations, inhibiting 65.92% of PR transactivation at the highest 400-fold concentration (Figure 1D), with a REQ of 16.45 ng/L water (Table 1). In GR assays, the OEs of tap water did not induce significant transactivation at all concentrations tested (Figure 1E), implying no detectable GR agonistic activity. However, the OEs attenuated HC-induced GR transactivation in a dose-dependent manner (Figure 1F), showing significant anti-glucocorticoid activity at 300- and 400-fold enrichment concentration, with a REQ value of 4.77 ng/L water (Table 1). In contrast to the results of the GR agonistic activity assay, the OEs of tap water significantly enhanced MR transactivation at a 50-fold enrichment concentration, peaking at a 100-fold concentration at 42.50% of the positive control ALD (Figure 1G), with an AEQ value for mineralocorticoid activity of 0.03 ng /L water (Table 1). Similar to the results of AR, PR, and GR antagonistic activity assays, the OEs reduced ALD-induced MR transactivation in a dose-dependent manner, which was statistically significant starting at 200-fold enrichment concentration, with a SEQ value of 138.81 ng/L water (Table 1).

Some previous studies from different countries and regions have also determined endocrine activity in drinking water using various in vitro bioassays, with ER agonistic and antagonistic activity assays being the most studied [16]. In agreement with our observations, a report from a city in northeast China showed that, instead of estrogenic activity, anti-estrogenic activity was detected in effluent samples from drinking water treatment plants (DWTPs) [41]. Another study showed that none of the drinking water samples of the central Spain exhibited estrogenic activities in in vitro luciferase reporter assays [42]. However, a few studies observed detectable estrogenic activity in drinking water. In a study from the Yangtze River Delta, China, Xiao et al. [43] reported negligible estrogenic activity in DWTP samples using yeast estrogen screen assays, with a median EEQ value of 0.38 ng/L water. By using reporter gene assays, both Rosenmai et al. [13] and Conley et al. [14] detected estrogenic activity in DWTPs from Sweden and USA, with a maximum concentration of 0.039 ng EEQ/L water and 0.078 ng EEQ/L water, respectively. The differences in bioassay responses between this study and some of the existing literature may be attributed to the differences in concentrations and types of chemicals in the source water and the variations in contaminant removal efficiencies of DWTP achieved by the diverse treatment technologies [44]. Additional contributing factors might include differences in cell line sensitivities, protein synthesis pathways, receptor affinities, and the cellular uptake of chemicals in the tested samples [45,46].

Compared to ER agonistic and antagonistic activity assays, the bioassays for PR, GR, and MR activities are applied relatively infrequently for the assessment of endocrine-disrupting effects in drinking water. Leusch et al. [47] determined the agonist and antagonist activity against ERα, PR, GR, and MR in the drinking water from six countries (Germany, Australia, France, South Africa, The Netherlands, and Spain), but the results showed no apparent endocrine risk to human health. A study from France demonstrated that no (ant)agonist activities for ERα, PR, and GR were detected in tap water [48]. Although few studies have yet reported MR activation and/or inhibition in drinking water, several investigations have identified detectable anti-mineralocorticoid activities in raw and treated wastewater as well as surface water [47,49,50].

In the agonistic assays against PR and MR in this study, the bar graphs of the OEs of tap water displayed an inverted U-shape, indicating an initial increase and subsequent decrease in agonistic activity as the enrichment factor increased (Figure 1). However, the antagonistic assays against ERα, PR, GR, and MR demonstrated a positive correlation between enrichment factor and antagonistic activity at higher concentrations (≥100 times) (Figure 1). Proportional models are inadequate for predicting agonistic or antagonistic responses when complex chemical mixtures are concentrated or diluted. These results provide insights into the complex interactions between endocrine disruptors in water and biological systems, warranting further investigation to identify these substances and evaluate their potential ecological and health impacts.

In this study, the absence of (anti-)estrogenic, (anti-)progesterone, (anti-)glucocorticoid, and (anti-)mineralocorticoid activity at enrichment concentrations of ≤10 times suggests that the tap water tested does not pose an immediate or acute health risk to humans. However, given the frequency and volume of water consumption by humans, the detection of endocrine activities at relatively higher concentrations still raises concerns about the potential health effects of long-term exposure to low levels of the mixture of compounds in drinking water [51]. Therefore, there is a need to strengthen efforts to mitigate the risks of biotoxicity. Because of the significant efficacy in assessing mixed pollutant toxicity and standardized operability, it is recommended that water quality monitoring agencies adopt bioassays like reporter gene assays to improve understanding of water pollution situations [13]. However, only a single tap water sample was used in this study, which limits the scope and applicability of the findings. Future studies should increase representativeness and diversity of the sample selection to improve the reliability and applicability of the results. Another limitation is that the endocrine-disrupting activity in procedural blank samples was not tested in this study. Thus, whether the extraction process induces significant endocrine-disrupting activity needs to be verified.

### 3.2. Effects of Boiling on Agonistic and Antagonistic Activity against ERα, PR, GR, and MR in Tap Water

**Figure 2:**
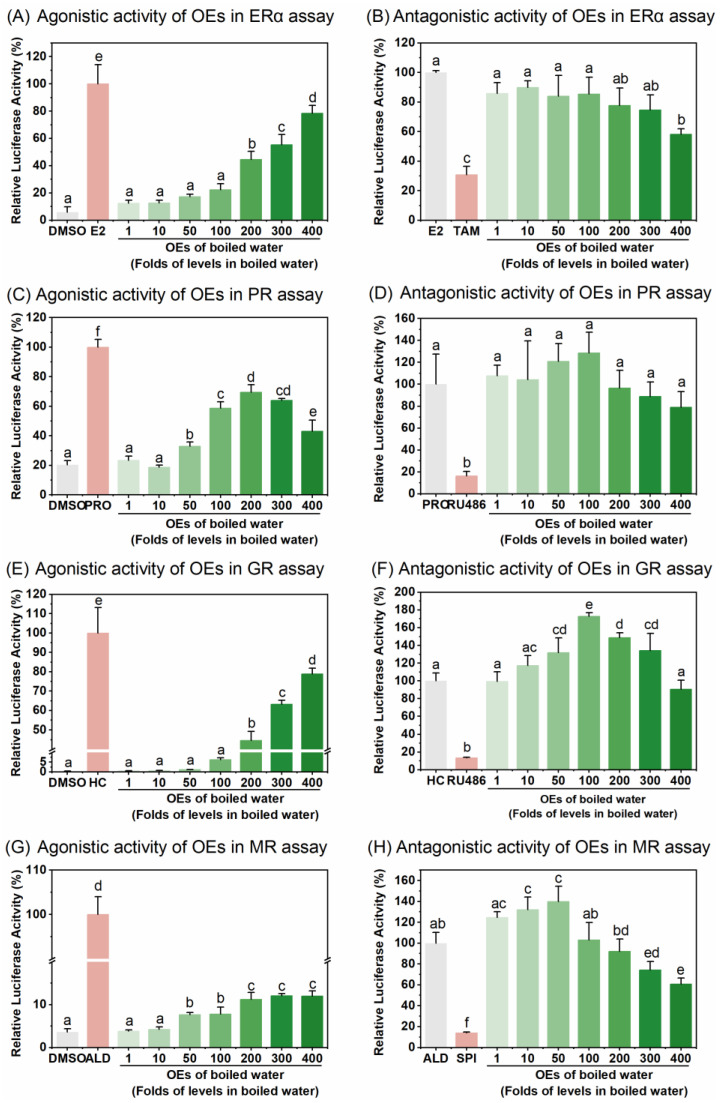
Agonistic and antagonistic activity of organic extracts (OEs) of boiled water in SHR bioassays. (**A**–**G**) The agonistic activities of OEs of boiled water in bioassays for ERα (**A**), PR (**C**), GR (**E**), and MR (**G**), respectively. (**B**–**H**) The antagonistic activities of OEs of boiled water in bioassays for ERα (**B**), PR (**D**), GR (**F**), and MR (**H**), respectively. Results were expressed as percent induction, with 100% activity defined as the activity achieved with positive control. Data are shown as mean ± SD (*n* = 4). Different letters on bars indicate significant differences among treatments (*p* < 0.05).

Boiling is considered to be a detoxification process and is the most commonly used method for treating water in China. In this study, the OEs of boiled tap water exhibited a concentration-dependent induction in estrogenic, progesterone, glucocorticoid, and mineralocorticoid activities that was statistically significant at relatively higher concentration levels, with agonistic activities of ER, PR, and GR reaching up to 69.58–78.84% of those of synthetic steroid hormones used as the positive controls (Figure 2). Comparing equivalent values of endocrine activity in boiled water and tap water (Table 1), boiling appeared to increase estrogenic and glucocorticoid activities from no detectable activity to 0.34 ng EEQ/L water and 3.22 ng HEQ/L water, respectively (Table 1), as well as decrease progesterone and mineralocorticoid activities from 43.84 ng PEQ/L to 29.51 ng PEQ/L, from 0.03 ng AEQ/L to 0.01 ng AEQ/L, respectively (Table 1). However, the antagonistic effects of OEs in boiled water on all four SHRs were less significant than that of tap water, with anti-estrogenic activity being significant only at the highest enriched concentration and no significant anti-progesterone or anti-glucocorticoid activity at all concentrations (Figure 2A). Only the anti-mineralocorticoid activity of OEs in boiled water showed a concentration-dependent increase. Compared with tap water, the anti-estrogenic, anti-progesterone, anti-glucocorticoid, and anti-mineralocorticoid activities of OEs in boiled water decreased from 4.53 μg TEQ/L to 3.67 μg TEQ/L, from 16.45 ng REQ/L to no detectable activity, from 4.77 ng REQ/L to no detectable activity, and from 138.81 ng SEQ/L to 54.55 ng SEQ/L, respectively (Table 1). The decrease in anti-steroid hormone activities after boiling in this study suggests possible degradation or structural modification of causative compounds that interact with these SHRs. A similar observation was reported in another study from China, where the anti-thyroid hormone receptor (TR) activity in some tap water samples no longer showed TR antagonistic activity after boiling [52].

However, this study showed that boiling increased estrogenic and glucocorticoid activities in OEs. The increased SHR agonistic activity in boiled water might result from the formation and release of compounds directly responsible for these bioactivities during boiling. Additionally, many organic pollutants such as pesticides or industrial chemicals that have higher boiling points than water may not evaporate or decompose easily during boiling. This could cause these chemicals to concentrate when water evaporates. Reactivity in bioassays due to water samples does not inherently indicate unacceptable water quality [53]. Brand et al. [54] proposed a recommended safety level of 3.80 ng EEQ/L water for an effect-based trigger value (EBT) for ERα agonistic activity in drinking water. The elevated EEQ value after boiling in this study remained below this threshold, indicating minimal health risks from the studied boiled water. However, the development and availability of EBTs are still limited. Although previous studies have explored EBTs for GR and PR agonistic activity in drinking water [53], their use of the reference agonists dexamethasone and levonorgestrel differ from this study and, therefore, direct comparisons with these values could not be made. Furthermore, EBTs regarding SHR antagonist activity for drinking water are limited, and there are currently no EBTs on mineralocorticoid activity. This gap highlights the need for further research to establish EBTs across a broader range of endocrine endpoints.

Studies on the effects of boiling on endocrine activity in OEs of tap water are limited. The different impacts of boiling on ERα, PR, GR, and MR observed in this study suggest that boiling alters the chemical composition of water, potentially leading to the formation or degradation of compounds that interact differently with SHRs. This observation is consistent with literature that suggests that thermal processes change the presence and efficacy of certain EDCs in water, particularly disinfection by-products [29,30,55] and phthalate esters [52,56]. It is important to understand these alterations and interactions because of their potential impact on public health, especially in areas where boiling is a common method of water purification.

### 3.3. Non-Target Analyses of Chemicals in Tap Water and Boiled Water Samples

**Figure 3:**
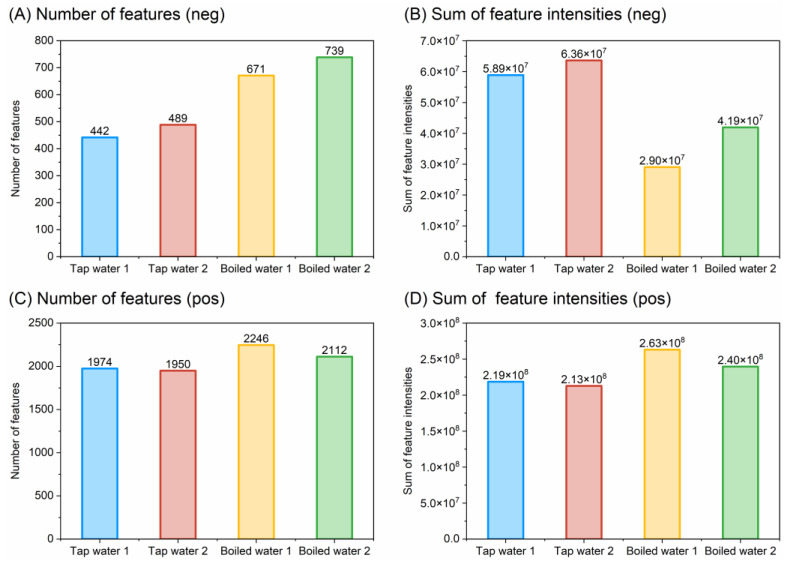
Number of features (**A**,**C**) and sum of feature intensities (**B**,**D**) detected per sample for negative (**A**,**B**) and positive (**C**,**D**) ionization.

In order to explore the potentially main contributing chemicals in tap water and boiled water that produce (anti-)steroid hormone activity and the effect of boiling on the composition of these substances, we further performed the non-target analysis of water samples. Two tap water samples and two boiled water samples were analyzed to ensure repeatability. Figure 3 and Figure S3 provide an overview of the detected features in water samples as well as detailed information on the *m*/*z* value, retention time, and intensity (reported as peak area) of the detected features in tap and boiled water. As shown in Figure 3A, in the negative ionization mode, boiled water samples exhibited a significantly higher count of feature numbers than the tap water samples. However, tap water samples had a higher sum of feature intensities than boiled water samples (Figure 3B). The increase in the number of features and decrease in signal intensities in the non-target features indicate a potential change in compound variety and a decrease in the total organic material mass after boiling. In the positive ionization mode (Figure 3C,D), boiled water samples had a greater number of features and higher sum of feature intensities than tap water samples. In all samples, positively ionizable compounds had more features than negatively ionizable ones. Notably, the chromatographic conditions in this study slightly favored positive ionization rather than negative ionization due to the use of formic acid in the mobile phase [57]. Integration of these chemical analyses with bioassay results reveals a lack of regularity in the direct correlations between the number of features or the sum of feature intensities and bioassay activity, suggesting the complexity of assessing water quality based solely on chemical analysis (Figure 3 and Table 1).

Among the detected features, 36 tentatively annotated structures were present in the MassBank list, while others were not found in spectral libraries. Analytical standards of tentatively identified compounds were acquired to confirm their identities. Of these compounds, only eight had commercially available reference standards and were, therefore, selected for further study. Based on the scale proposed by Schymanski et al. [58], analysis of reference standards confirmed the identities of DNP, 12-HA, and 3-HTA (level 1), with MS and MS/MS spectrum shown in Figures S4–S6. The other five compounds, i.e., DBSA, ISO, PBSA, 2-NSA, and BEN, were assigned identification confidence level 2a (accurate mass spectral library match) and the supporting evidence was shown in Figures S7–S11.

### 3.4. Endocrine-Disrupting Effects of the (Tentatively) Identified Chemicals

**Figure 4:**
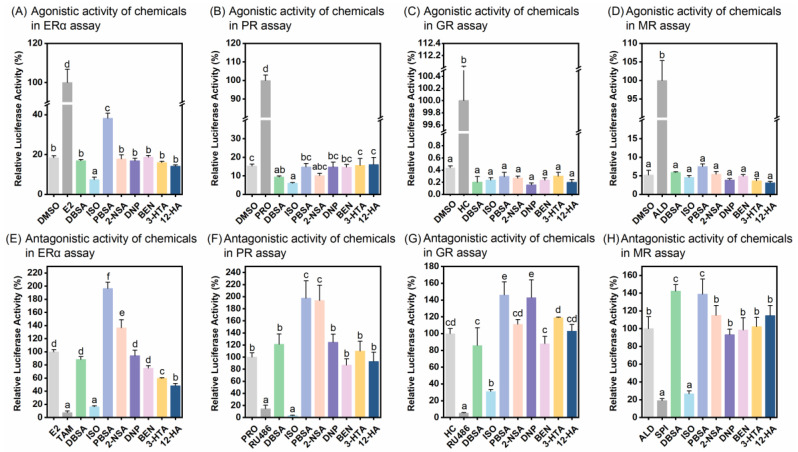
Agonistic and antagonistic activity of eight (tentatively) identified chemicals in bioassays for ERα, PR, GR, and MR. (**A**–**D**) The agonistic activities of eight chemicals at their corresponding highest noncytotoxic concentrations (10^−6^ M of DBSA, 10^−5^ M of ISO, 10^−4.5^ M of PBSA, 10^−4^ M of 2-NSA, 10^−7^ M of DNP, 10^−5^ M of BEN, 10^−4^ M of 3-HTA, and 10^−4^ M of 12-HA) in bioassays for ERα (**A**), PR (**B**), GR (**C**), and MR (**D**), respectively. (**E**–**H**) The antagonistic activities of eight chemicals at their corresponding highest noncytotoxic concentrations in bioassays for ERα (**E**), PR (**F**), GR (**G**), and MR (**H**), respectively. Results were expressed as percent induction, with 100% activity defined as the activity achieved with positive control. Data are shown as mean ± SD (*n* = 4). Different letters on bars indicate significant differences among treatments (*p* < 0.05).

The endocrine activities of eight (tentatively) identified compounds against ERα, PR, GR, and MR were further determined at their highest non-cytotoxic concentrations (Figure 4, Table 2). Of the four SHR agonist activity assays, among the eight chemicals, only PBSA showed significant estrogenic activity (Figure 4A). Even in the ERα antagonistic activity assay, PBSA significantly enhanced E2-induced ERα transactivity, indicating a synergistic effect (Figure 4E). Of the eight compounds, ISO showed a strong antagonistic effect on all four SHRs, inhibiting 68.80–96.90% of the SHR transactivities compared to the steroid controls (Figure 4). 3-HTA and 12-HA also exhibited significant anti-estrogenic activity (Figure 4E). Although 2-NSA, DNP, and DBSA enhanced steroid hormone-induced SHR activity in the antagonistic activity assays, they had no significant effects in the corresponding SHR agonistic activity assays, suggesting that the steroid-like activities of these compounds were too weak when they acted individually at the concentrations tested and only exhibited synergistic effects when co-exposed with steroid hormones (Figure 4). BEN showed neither agonistic nor antagonistic activity in any SHR assays (Figure 4).

**Figure 5:**
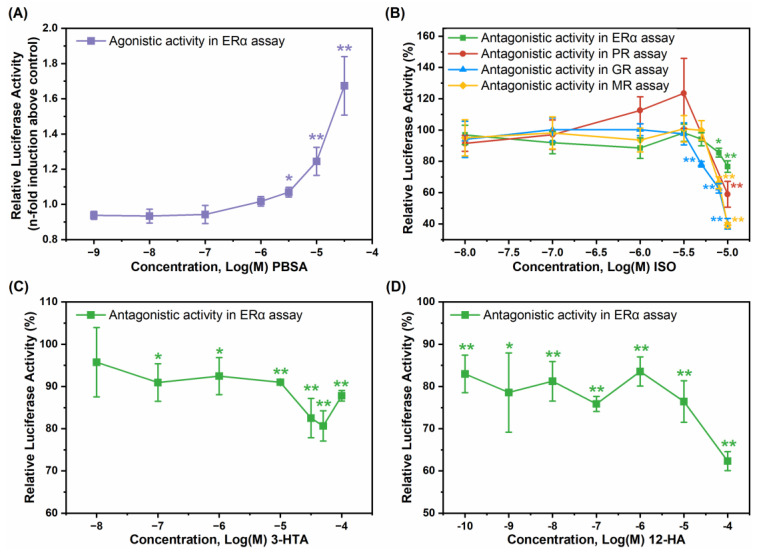
Dose–response agonist and antagonist activity of four (tentatively) identified chemicals in ERα, PR, GR, and MR assays. (**A**) The dose–response agonist activity of PBSA in ERα assay. (**B**) The dose–response antagonist activity of ISO in ERα, PR, GR, and MR assays. (**C**) The dose–response antagonist activity of 12-HA in ERα assay. (**D**) The dose–response antagonist activity of 3-HTA in ERα assay. Results were expressed as fold induction above negative control or percent induction, with 100% activity defined as the activity achieved with positive control. Data are shown as mean ± SD (*n* = 4). * indicates *p* < 0.05 and ** indicates *p* < 0.01, compared with control.

The concentration-dependent endocrine activities of four active compounds were further determined, including the ERα agonistic activity of PBSA at concentrations of 10^−9^–10^−4.5^ M (Figure 5A), the antagonistic activities of ISO against ERα, PR, GR, and MR at concentrations of 10^−8^–10^−5^ M (Figure 5B), as well as the ERα antagonistic activity of 10^−8^–10^−4^ M 3-HTA and 10^−10^–10^−4^ M 12-HA (Figure 5C,D). The 1.2-fold relative activation concentration (RAC_1.2_) of PBSA, i.e., the concentration at which ERα transactivation is induced to be 1.2-fold compared to DMSO (negative control), was estimated from the concentration-response curve. The 20% and 50% relative inhibitory concentrations (RIC_20_ and RIC_50_), representing the concentrations at which the test chemicals reduce agonist-induced SHR transactivation by 20% and 50%, respectively, were estimated based on the concentration-response curves for 3-HTA, 12-HA, and ISO (Table 2). The relative inhibition rate (RIR) and relative activation fold (RAF) of these chemicals at their highest tested concentrations, reflecting the percent decrease of agonist or fold increase of DMSO responses, are also given in Table 2. PBSA showed concentration-dependent estrogenic activity, with a RAC_1.2_ value of 8.27 × 10^−6^ M and an RAF value of 1.67 (Figure 5A, Table 2). ISO had comparable anti-estrogenic and anti-progesterone activities with RIC_20_ values of 9.99 × 10^−6^ M and 8.15 × 10^−6^ M, respectively, and potent antagonistic activities against GR and MR with inhibition rates surpassing 50% and RIC_50_ values of 1.00 × 10^−5^ M and 1.21 × 10^−5^ M, respectively (Figure 5B, Table 2). 12-HA exhibited significantly higher anti-estrogenic potency than 3-HTA, with a RIC_20_ value of 6.92 × 10^−10^ M (Table 2), while the anti-estrogenic activity of 3-HTA was weaker, with a maximum inhibition rate not exceeding 20% (Figure 5C,D, Table 2).

**Figure 6:**
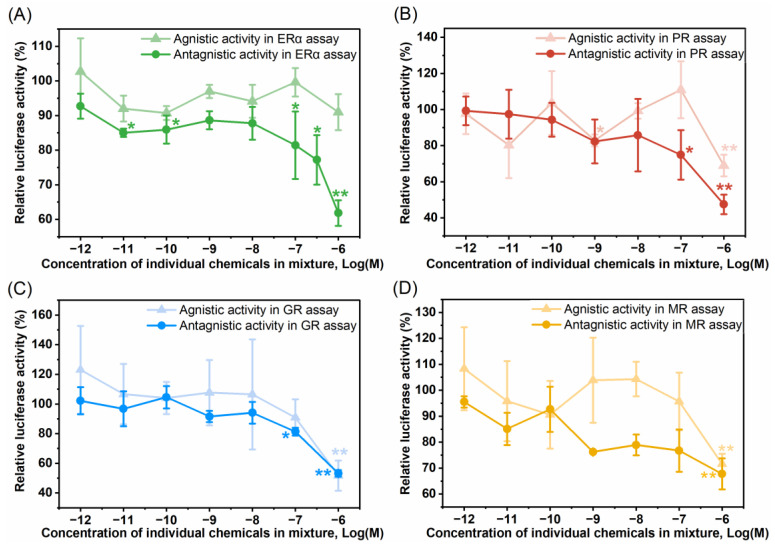
Dose–response agonist and antagonist activity of the mixture of eight (tentatively) identified chemicals with an equal individual chemical concentration of 10^−12^ to 10^−6^ M in SHR bioassays. (**A**–**D**) The agonistic and antagonistic activities of the mixture of eight chemicals in bioassays for ERα (**A**), PR (**B**), GR (**C**), and MR (**D**), respectively. Results were expressed as percent induction, with 100% activity defined as the activity achieved with control. Data are shown as mean ± SD (*n* = 4). * indicates *p* < 0.05 and ** indicates *p* < 0.01, compared with control.

Because of coexistence of these chemicals in the tap water, the agonist and antagonist activities of a mixture of the eight (tentatively) identified chemicals at their individual concentration of 10^−12^–10^−6^ M, were examined in SHR assays (Figure 6). The mixture had no significant agonistic activity against the four SHRs at all concentrations tested, and even inhibited the transactivation of the SHRs at the highest concentration (Figure 6). Although PBSA had significant estrogenic activity when exposed alone, the mixture had no estrogenic activity, probably because the other compounds antagonized the action of PBSA. The combination of these eight chemicals appeared to produce a synergistic effect in antagonistic activities against SHRs, significantly attenuating steroid hormone-induced SHR transactivation at relatively low concentrations (Figure 6). The maximum RIRs in each assay were relatively higher than the sum of RIRs for these individual chemicals at the concentration of 10^−6^ M (Table 2).

BEN is a commonly used benzothiadiazole herbicide that has been detected in tap water and surface water in China, groundwater in Europe, and effluent from ditches in France, with the maximal concentrations ranging from 0.047 μg/L to 11 μg/L [2,59,60,61]. A previous study using yeast screen reported that BEN had anti-androgenic activity at concentrations of 500–1000 μM, but no (anti-)estrogenic activity as well as androgenic activity [62], the latter of which is consistent with our observation that BEN had no agonistic or antagonistic activity against ER. PBSA is a water-soluble UV filter widely used in sunscreens. PBSA has been detected in a variety of aquatic environments, such as in tap water in Spain at 10 ng/L [63], in surface water in Milan at 560.4 ng/L [64], and in Australian wastewater treatment plants at 4.49 μg/L [65]. A recent study found that PBSA disrupted plasma sex steroid hormone levels and altered the expression levels of genes in hypothalamic–pituitary–gonadal axis in zebrafish, suggesting an in vivo endocrine-disrupting effect of PBSA [66]. This study demonstrated for the first time that PBSA interacted with ERα and showed significant estrogenic activity. The presence of PBSA as an estrogen-like hormone in drinking water may pose a potential risk to human health.

To our best knowledge, six of the eight substances identified in this study, i.e., ISO, 3-HTA, 12-HA, DNP, DBSA, and 2-NSA, are reported to be detected in tap water for the first time. We found for the first time that ISO, 3-HTA, and 12-HA that are natural metabolites of bacteria, plants or humans, had antagonistic activities against SHRs, and in particular, ISO showed potent antagonistic effects against all four SHRs tested. ISO is a natural product found in plant *Salvia pachyphylla* and is considered as an environmentally hazardous substance that is toxic to aquatic life with both acute and long-lasting effects [67]. 3-HTA is a long-chain fatty acid, a bacterial metabolite and human metabolite that has been used as an environmental marker to detect endotoxins such as those found in dialysis water [68,69]. 12-HA is a hydroxy fatty acid, a plant metabolite and bacterial xenobiotic metabolite that can be used as a gelator and co-surfactant in surfactant-containing systems [70]. These three chemicals may also pose a potential risk to human health due to their endocrine-disrupting activity when they enter drinking water.

2-NSA and DBSA are industrial organic chemicals. 2-NSA has a wide range of uses as a monomer in thermoplastic manufacture, intermediate for large-scale chemicals, and plastifier for concrete mixtures [71]. DBSA is a surfactant utilized in the production of detergents [72]. DNP is an antioxidant derivative formed during the combustion of diesel fuel containing antioxidants such as 2,6-di-tert-butylphenol, and has been detected in European surface waters at an average concentration of 17 ng/L [73,74]. Although these three substances had no significant (ant)agonistic activities against SHRs when acting alone, it is noteworthy that the mixture of 8 chemicals containing them exhibited stronger antagonistic activities against SHRs. Therefore, more attention and research should be given to the endocrine-disrupting effects and health risks of chemical mixtures in drinking water.

## 4. Conclusions

Although the tap water tested in this study does not pose an immediate or acute health risk to humans, the potential health effects still raise concerns due to endocrine activities detected in tap water at relatively higher enrichment concentrations. We found that boiling decreased anti-estrogenic, (anti-)progesterone, anti-glucocorticoid, and (anti-)mineralocorticoid activities in tap water, suggesting boiling as an effective method for reducing certain potential anti-steroid hormone activities in drinking water. However, we also found for the first time that boiling elevated estrogenic and glucocorticoid activities in tap water.

Notably, this study identified four novel potential EDCs, i.e., PBSA, 3-HTA, 12-HA, and ISO, by non-target analysis and in vitro bioassays. The interaction of identified chemicals suggests the synergistic effects in antagonistic activities against SHRs, thus complicating the understanding of the overall health risks of combined pollution to humans. The lack of appropriate internal standards for some (tentatively) identified chemicals poses a challenge to the accurate monitoring of their concentrations in drinking water and further hinders the verification of bioassay results against the known endocrine activities of these chemicals. Thus, despite uncertainties in the type, amount, and density of chemicals, the application of rapid in vitro bioassays to determine the total endocrine-disrupting effects of mixtures extracted from drinking water samples may provide a more adequate overall assessment of the potential health risks of drinking water contamination.

## Figures and Tables

**Table 1:** Agonistic and antagonistic equivalents for ERα, PR, GR, and MR in water.

	ERα		PR		GR		MR	
	EEQ (ng/L)	TEQ (μg/L)	PEQ (ng/L)	REQ (ng/L)	HEQ (ng/L)	REQ (ng/L)	AEQ (ng/L)	SEQ (ng/L)
Tap water	-	4.53	43.84	16.45	-	4.77	0.03	138.81
Boiled water	0.34	3.67	29.51	-	3.22	-	0.01	54.55

E_2_ equivalent (EEQ) for ERα agonistic activity, TAM equivalent (TEQ) for ERα antagonistic activity, PRO equivalent (PEQ) for PR agonistic activity, RU486 equivalent (REQ) for PR and GR antagonistic activity, HC equivalent (HEQ) for GR agonistic activity, ALD equivalent (AEQ) for MR agonistic activity, SPI equivalent (SEQ) for MR antagonistic activity. - represents no significant activity detected.

**Table 2:** Lists of 8 (tentatively) identified chemicals and a mixture of the eight chemicals, their identification confidence level (ICL), the highest noncytotoxic concentration (HNC), and bioactivity.

Compound	Formula	ICL	HNC (M)	Bioactivity					
				Endpoints	RIC_20_ (M)	RIC_50_ (M)	RIR (%)	RAC_1.2_ (M)	RAF
Dodecylbenzene sulfonic acid (DBSA)	C18H30O3S	2a	10^−6^	NA	NA	NA	NA	NA	NA
Isorosmanol (ISO)	C20H26O5	2a	10^−5^	ERα antagonistic activity	9.99 × 10^−6^	NA	23.34	NA	NA
				PR antagonistic activity	8.15 × 10^−6^	NA	41.00	NA	NA
				GR antagonistic activity	5.07 × 10^−6^	1.00 × 10^−5^	59.90	NA	NA
				MR antagonistic activity	7.71 × 10^−6^	1.21 × 10^−5^	60.78	NA	NA
Phenylbenzimidazole sulfonic acid (PBSA)	C13H10N2O3S	2a	10^−4.5^	ERα agonistic activity	NA	NA	NA	8.27 × 10^−6^	1.67
2-Naphthalene sulfonic acid (2-NSA)	C10H8O3S	2a	10^−4^	NA	NA	NA	NA	NA	NA
2,6-Di-tert-butyl-4-nitrophenol (DNP)	C14H21NO3	1	10^−7^	NA	NA	NA	NA	NA	NA
Bentazone (BEN)	C10H12N2O3S	2a	10^−5^	NA	NA	NA	NA	NA	NA
beta-Hydroxymyristic acid (3-HTA)	C14H28O3	1	10^−4^	ERα antagonistic activity	NA	NA	12.19	NA	NA
12-Hydroxyoctadecanoic acid (12-HA)	C18H36O3	1	10^−4^	ERα antagonistic activity	6.92 × 10^−10^	NA	37.67	NA	NA
Mixture ^a^	NA	NA	10^−6^	ERα antagonistic activity	NA	NA	38.17	NA	NA
				PR antagonistic activity			52.52		
				GR antagonistic activity			46.77		
				MR antagonistic activity			32.25		

^a^ the mixture of the eight chemicals at their individual concentration of 10^−6^ M. NA: not available. RIC_20_: concentration of tested chemicals that inhibited 20% of the luciferase activity induced by corresponding agonists. RIC_50_: concentration of tested chemicals that inhibited 50% of the luciferase activity induced by corresponding agonists. RIR: relative inhibition rate obtained at highest test concentration of chemicals as percent decrease of corresponding agonists response. RAC_1.2_: concentration of tested chemicals that induced 1.2-fold of the luciferase activity, compared to negative control. RAF: relative activation fold obtained at highest test concentration of chemicals as fold increase of negative control response.

## Data Availability

Data is contained within article and Supplementary Materials.

## Supplementary Materials

The following supporting information can be downloaded at: https://www.mdpi.com/article/10.3390/toxics12040247/s1, Figure S1: Violin plot of recovery rates of 43 external standards. Figure S2: The viability of CHO-K1 cells exposed to OEs from tap water without or with boiling. Figure S3: Plots of *m*/*z* and retention time of the non-target features detected per sample for negative and positive ionization. Figure S4: MS and MS/MS spectrum of DNP (precursor *m*/*z* 250.1) identified in the water sample. Figure S5: MS and MS/MS spectrum of 3-HTA (precursor *m*/*z* 243.2) identified in the water sample. Figure S6: MS and MS/MS spectrum of 12-HA (precursor *m*/*z* 299.3) identified in the water sample. Figure S7: Spectral similarity of *m*/*z* 325.184 [M-H]^−^ to MassBank spectrum of DBSA. Figure S8: Spectral similarity of *m*/*z* 345.168 [M-H]^−^ to MassBank spectrum of ISO. Figure S9: Spectral similarity of *m*/*z* 273.033 [M-H]^−^ to MassBank spectrum of PBSA. Figure S10: Spectral similarity of *m*/*z* 207.011 [M-H]^−^ to MassBank spectrum of 2-NSA. Figure S11: Spectral similarity of *m*/*z* 239.049 [M-H]^−^ to MassBank spectrum of BEN. Table S1: Information on 43 external standards. References [11,75,76,77,78,79,80,81,82,83,84,85,86,87] are cited in the Supplementary Material.
