# Supplementary material for: An Integrated Approach of Bioassays and Non-Target Screening for the Assessment of Endocrine-Disrupting Activities in Tap Water and Identification of Novel Endocrine-Disrupting Chemicals
Source: Toxics. 2024 Mar 28;12(4):247. doi: 10.3390/toxics12040247 (PMC11054029; doi:10.3390/toxics12040247)
Supplement: Supplementary file 1 [file toxics-12-00247-s001.zip › toxics-2902737-supplementary.pdf]

toxics

*Supplementary materials*

# An Integrated Approach of Bioassays and Non-Target Screening for the Assessment of Endocrine-Disrupting Activities in Tap Water and Identification of Novel Endocrine-Disrupting Chemicals

**Siyuan Liu [1,2] and Jing Liu [1,2,*]**

**Table S1.** Information on 43 external standards.

| Name | Formula | CAS number | Reported bioactivity | References |
|---|---|---|---|---|
| Diethyl phthalate | C12H14O4 | 84-66-2 | ER↑ | [75] |
| Dibutyl phthalate | C16H22O4 | 84-74-2 | ER↑, ER↓ | [76, 77] |
| Diisobutyl phthalate | C16H22O4 | 84-69-5 | ER↑ | [76] |
| Dipentyl phthalate | C18H26O4 | 131-18-0 | ER↑, ER↓ | [76] |
| Dioctyl phthalate | C24H38O4 | 117-84-0 | ER↑ | [78] |
| Dinonyl phthalate | C26H42O4 | 84-76-4 | ER↑ | [78] |
| Dicyclohexyl phthalate | C20H26O4 | 84-61-7 | ER↑, ER↓ | [76] |
| Diphenyl phthalate | C20H14O4 | 84-62-8 | - | |
| Benzyl butyl phthalate | C19H20O4 | 85-68-7 | ER↑, ER↓ | [76, 77] |
| Dimethylglycol phthalate | C14H18O6 | 117-82-8 | - | |
| Diethoxyethyl phthalate | C16H22O6 | 605-54-9 | - | |
| Bis(2-butoxyethyl) phthalate | C20H30O6 | 117-83-9 | - | |
| Pentachlorophenol | C6HCl5O | 87-86-5 | ER↑, ER↓, PR↓ | [79] |
| Tetrabromobisphenol A | C15H12Br4O2 | 79-94-7 | ER↑, PR↓ | [79] |
| Phenol | C6H6O | 108-95-2 | - | |
| Parathion-methyl | C8H10NO5PS | 298-00-0 | - | |
| Malathion | C10H19O6PS2 | 121-75-5 | ER↑, GR↓ | [80, 81] |
| Dimethoate | C5H12NO3PS2 | 60-51-5 | ER↑, MR↓ | [82, 83] |
| Bentazone | C10H12N2O3S | 25057-89-0 | - | |
| Perfluoropentanoic acid | C5HF9O2 | 2706-90-3 | - | |
| Perfluorohexanoic acid | C6HF11O2 | 307-24-4 | - | |
| Perfluoroheptanoic acid | C7HF13O2 | 375-85-9 | - | |
| Pentadecafluorooctanoic acid | C8HF15O2 | 335-67-1 | ER↑ | [84] |
| Nonafluorobutane-1-sulfonic acid | C4HF9O3S | 375-73-5 | - | |
| Perfluorohexane-1-sulphonic acid | C6HF13O3S | 355-46-4 | ER↑ | [85] |
| Perfluoroheptanesulfonic acid | C7HF15O3S | 375-92-8 | - | |
| Perfluorooctanesulfonic acid | C8HF17O3S | 1763-23-1 | ER↑ | [85] |
| Imidacloprid | C9H10ClN5O2 | 138261-41-3 | ER↑ | [86] |
| Imidaclothiz | C7H8ClN5O2S | 105843-36-5 | - | |
| Thiamethoxam | C8H10ClN5O3S | 153719-23-4 | - | |
| Clothianidin | C6H8ClN5O2S | 210880-92-5 | - | |
| Thiacloprid | C10H9ClN4S | 111988-49-9 | ER↑ | [86] |
| Acetamiprid | C10H11ClN4 | 135410-20-7 | - | |
| Dinotefuran | C7H14N4O3 | 165252-70-0 | - | |
| Nitenpyram | C11H15ClN4O2 | 150824-47-8 | - | |
| Thiacloprid-amide | C10H11ClN4OS | 676228-91-4 | - | |
| N-desmethyl-acetamiprid | C9H9ClN4 | 190604-92-3 | - | |
| Flonicamid | C9H6F3N3O | 158062-67-0 | - | |
| Benzo[a]pyrene | C20H12 | 50-32-8 | ER↑, ER↓, PR↑, GR↓ | [87] |
| Benzo[b]fluoranthene | C20H12 | 205-99-2 | - | |
| Benzo[k]fluoranthene | C20H12 | 207-08-9 | - | |
| 3-phenoxy benzoic acid | C13H10O3 | 3739-38-6 | GR↓ | [11] |
| 4-fluoro-3-phenoxy benzoic acid | C13H9FO3 | 77279-89-1 | - | |

All external standards were obtained from Tan-Mo Technology Co., Ltd. (Changzhou, China). ER: estrogen receptor. PR: progesterone receptor. GR: glucocorticoid receptor. MR: mineralocorticoid receptor. ↑: agonistic activity. ↓: antagonistic activity. - represents no agonistic or antagonistic activity in ER, PR, GR, or MR assays reported in the literature.

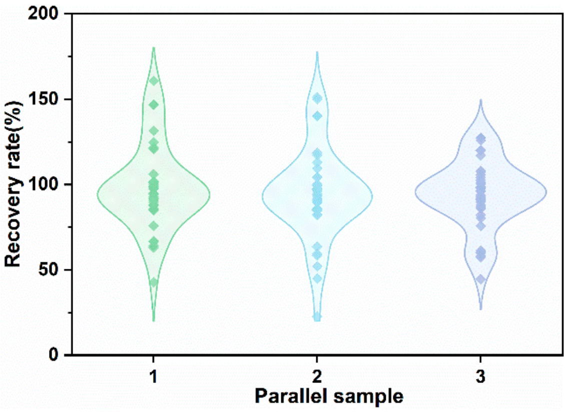


**Figure S1.** Violin plot of recovery rates of 43 external standards.

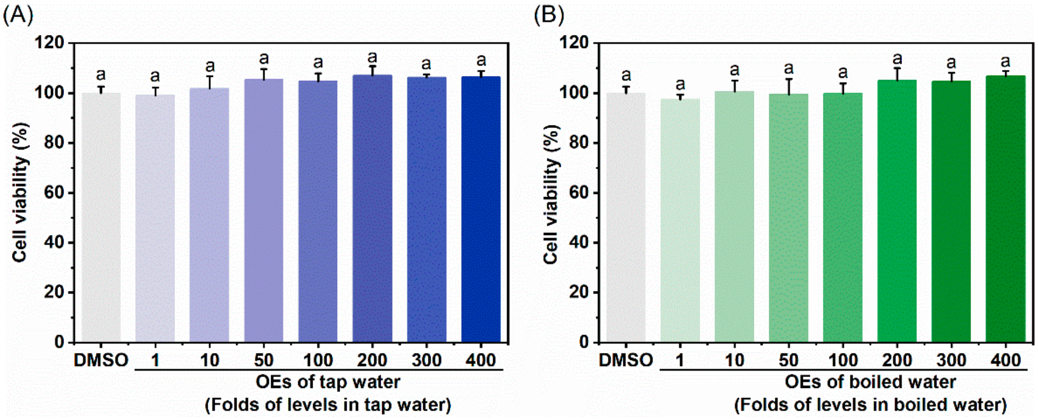


**Figure S2.** The viability of CHO-K1 cells exposed to OEs from tap water without or with boiling. **(A)** The cytotoxicity of OEs of tap water to CHO-K1 cells. CHO-K1 cells were exposed to the OEs of tap water or 1% DMSO (as vehicle control) for 24h. **(B)** The cytotoxicity of OEs of boiled water to CHO-K1 cells. CHO-K1 cells were exposed to the OEs of boiled water or 1% DMSO (as vehicle control) for 24h. Results were expressed as percent of control. Data are shown as mean±SD (n=6). Different letters on bars indicate significant differences among treatments ($p$<0.05).

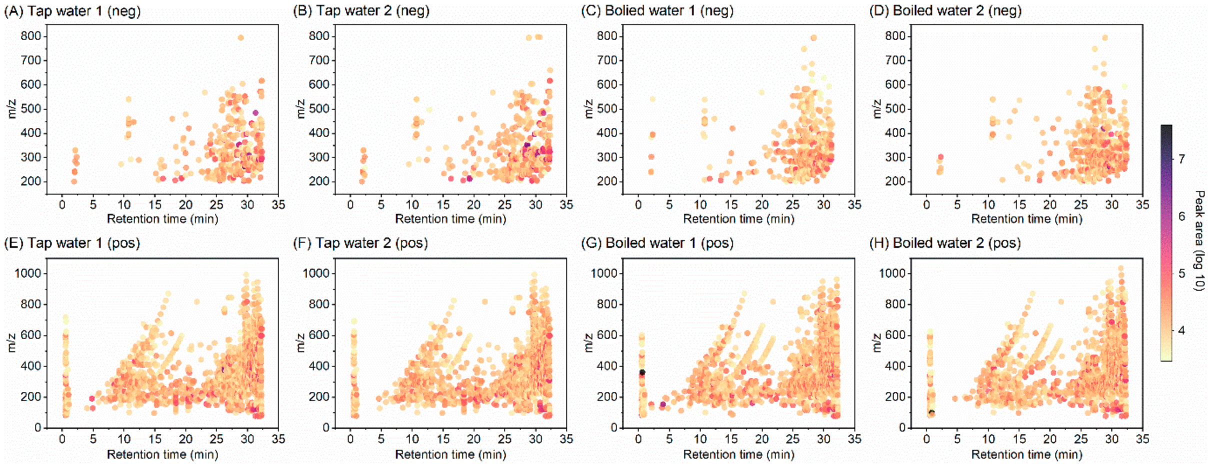


**Figure S3.** Plots of *m*/*z* and retention time of the non-target features detected per sample for negative **(A, B, C, D)** and positive **(E, F, G, H)** ionization. The color presents the peak area of a certain feature.

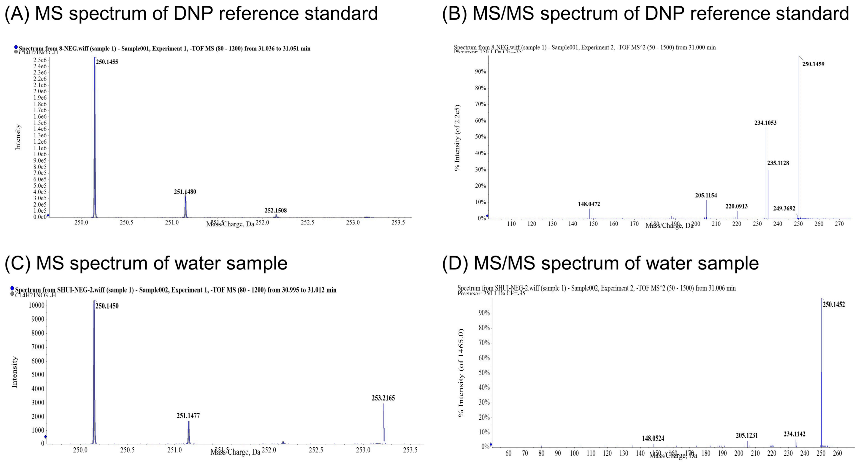


**Figure S4** MS and MS/MS spectrum of DNP (precursor *m*/*z* 250.1) identified in the water sample.

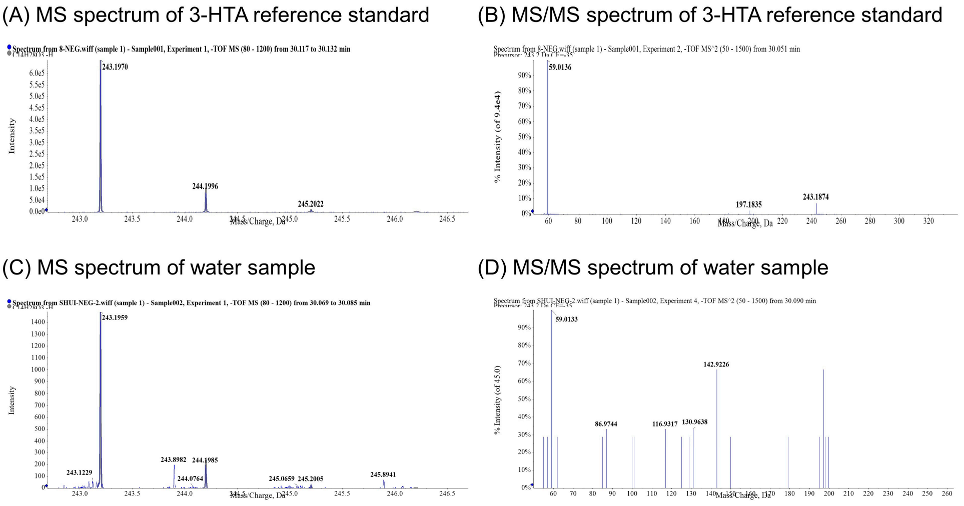


**Figure S5** MS and MS/MS spectrum of 3-HTA (precursor *m*/*z* 243.2) identified in the water sample.

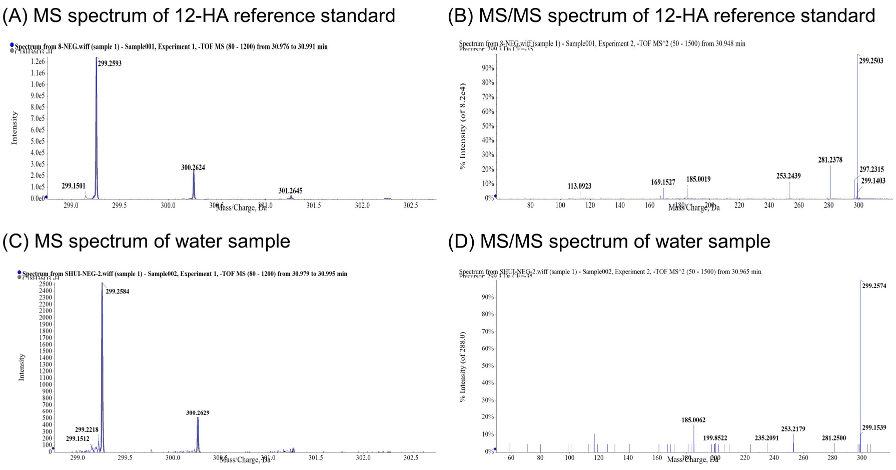


**Figure S6** MS and MS/MS spectrum of 12-HA (precursor m/z 299.3) identified in the water sample.

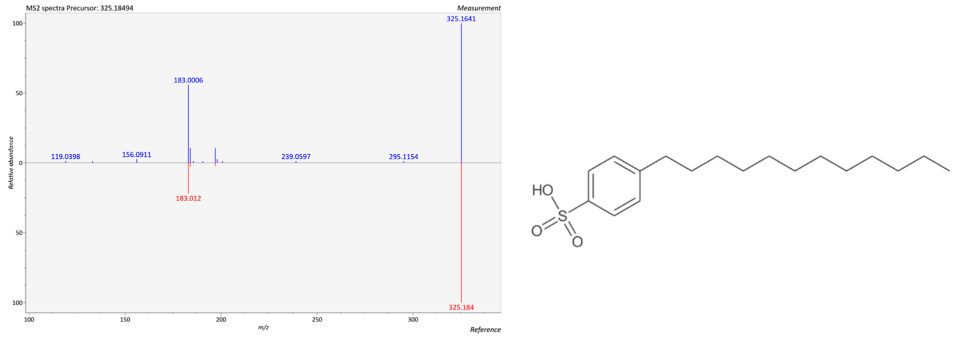


**Figure S7** Spectral similarity of *m/z* 325.184 [M-H]- to MassBank spectrum of DBSA.

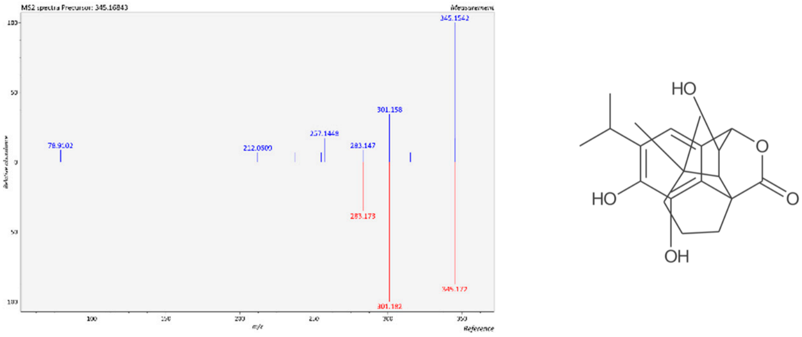


**Figure S8** Spectral similarity of *m/z* 345.168 [M-H]- to MassBank spectrum of ISO.

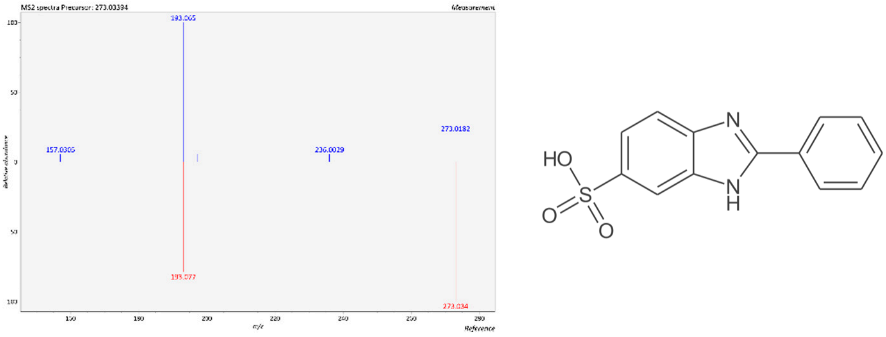


**Figure S9** Spectral similarity of *m/z* 273.033 [M-H]- to MassBank spectrum of PBSA.

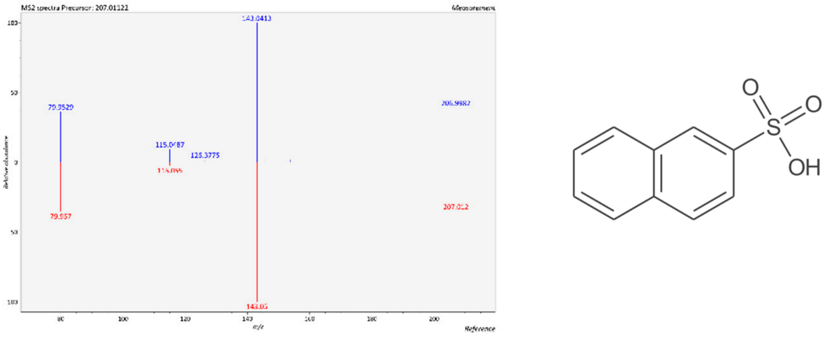


**Figure S10** Spectral similarity of *m/z* 207.011 [M-H]- to MassBank spectrum of 2-NSA.

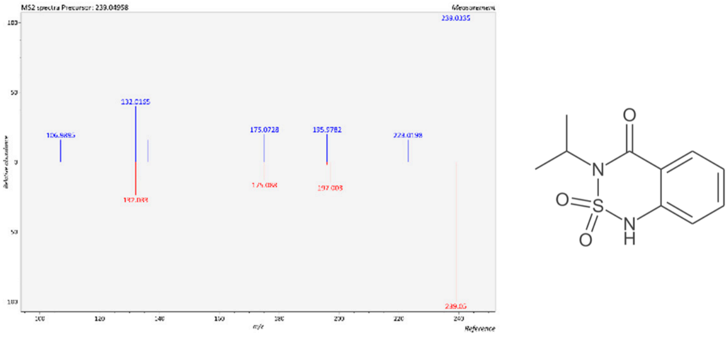


**Figure S11** Spectral similarity of *m/z* 239.049 [M-H]$^-$ to MassBank spectrum of BEN.
